# Death Quickly Produced by the Inhalation of Two Drachms of Chloloform, in the First Stage of Natural Labor, in Scotland

**Published:** 1859-02

**Authors:** Robert Lee

**Affiliations:** Obstetric Physician to St. George’s Hospital


*Death quickly produced by the Inhalation of two Drachms of Chloroform, in the first stage of Natural Labor, in Scotland.* By ROBERT LEE, M. D., F. R. S., Obstetric Physician to St. George's Hospital.

During the night of the 10th ult., while attending a lady in labor, a relative of whom was a warm advocate for the use of chloroform, the husband informed me that a fatal case had just occurred in Ayrshire, close to the residence of his brother, and that Mrs. B. had been attended by Dr. Campbell, of Largs. I immediately wrote to Dr. C., and received from him the following most satisfactory and polite answer. A few weeks only have elapsed since it was publicly denied that any case of death from chloroform during labor had ever occurred in Scotland :

LARGS, Ayrshire, Oct. 27, 1858.

DEAR SIR—I had the pleasure of receiving your note of the 23d inst., yesterday morning. I had been in the habit of attending Mrs. —— since January, 1850. She lived at Wemyss Bay, a distance of upwards of six miles from Largs. Mrs. —— was then pregnant for the first time. During the whole of February she had repeated attacks of hemorrhage from placenta prævia. On the 2d of March, labor came on, accompanied with hemorrhage, and as soon as the os was sufficiently dilated, I put her under chloroform, and delivered her by turning. This was most successfully performed so far as the patient was concerned, but the baby was still-born. Since that period she has been six times pregnant, and she had chloroform at each of her confinements; at least I am told so, for at two of these labors I did not arrive in time to witness delivery.

It is not my practice to give chloroform in natural, easy labors. I think it justifiable only in cases of unusual suffering, or where painful manual assistance is necessary : but Mrs. ——— having experienced the comfort of exemption from pain, and no unpleasant result from the use of it, insisted on having chloroform and her husband would give it. When I was present I took care that it was sparingly and cautiously given, and, as it happened, always with a satisfactory result.

On the occasion of her last and fatal labor, I understood I was to be called as usual ; but for some reason not very satisfactorily explained, I was not sent for. I had made a friendly call upon her on the 15th September, and found her pretty well and in good spirits. Her time was then up ; and her nurse, who is a midwife of considerable experience, had been with her from the 4th. On the morning of the 20th I had occasion to go to Wemyss bay to visit a patient, and I landed at the pier at ten minutes past eight A. M. I was met by a servant of Mrs. ———, who told me that she was alarmingly ill, and begged me to go to her without delay. I went directly, *and you may guess my horror when I found her stretched lifeless on the bed!* She had been dead about ten minutes : I spent about half an hour in fruitless attempts at reanimation. I was told that she begun to complain at two o'clock, and had been moving about and very cheerful all the morning. About twenty minutes to eight, expulsive pains came on, when she called for the chloroform; on giving it probably for the fourth time, *she threw herself violently back, gave a gasp or two; a slight gurgle was heard in her throat, and respiration and the pulse instantly ceased.*

The head was resting on the perineum. If I had had forceps at hand I might have brought away the child easily; but, as a matter of course, the cry was, 'can nothing be done to save the mother?' and after these attempts it was useless to effect delivery. The quantity of chloroform given in all probability did not exceed two drachms. The bottle from which it was taken could not have held more than an additional half ounce, and it was not full when Mr. ——— began to administer it.

I applied for a post-mortem examination, but it was declined.

Mrs. B. was a tall, thin person, who always during the married life was in delicate health. I was not acquainted with her before her marriage. She suffered from indigestion, and was unable to take any considerable amount of exercise; nor could she nurse any of her children. In July last she had a feverish attack, and a decided threatening of premature labor, accompanied with some sanguineous discharge, and from that time her pulse was always unnaturally full and frequent.

If I do not ask too great a favor, it would oblige me if you would favor me with any remarks or suggestions which occur to you relative to this distressing case, and also your opinion on the propriety of using chloroform in the practice of midwifery.

I am, &c.,

JOHN CAMPBELL.

P. S.—The chloroform was given on a common muslin handkerchief.—J. C."

